# Development of a high sensitivity TaqMan-based PCR assay for the specific detection of *Mycobacterium tuberculosis* complex in both pulmonary and extrapulmonary specimens

**DOI:** 10.1038/s41598-018-33804-1

**Published:** 2019-01-14

**Authors:** Hsin-Yao Wang, Jang-Jih Lu, Ching-Yu Chang, Wen-Pin Chou, Jason Chia-Hsun Hsieh, Chien-Ru Lin, Min-Hsien Wu

**Affiliations:** 1 Department of Laboratory Medicine, Chang Gung Memorial Hospital at Linkou, Taoyuan City, Taiwan; 2 Ph.D. Program in Biomedical Engineering, Chang Gung University, Taoyuan City, Taiwan; 3 Department of Medical Biotechnology and Laboratory Science, Chang Gung University, Taoyuan City, Taiwan; 4 School of Medicine, Chang Gung University, Taoyuan city, Taiwan; 5 Graduate Institute of Biomedical Engineering, Chang Gung University, Taoyuan city, Taiwan; 6 Taiwan/Division of Haematology/Oncology, Department of Internal Medicine, Chang Gung Memorial Hospital at Linkou, Taoyuan City, Taiwan

## Abstract

Tuberculosis (TB) causes a heavy health burden worldwide, especially in developing countries. The need for the rapid and accurate diagnosis of TB has not been satisfied, especially for extra-pulmonary specimens or specimens with acid fast stain (AFS)-negative condition. Development and validation of a novel, sensitive and specific assay for diagnosing TB is essential. We developed IS4 primer/probe based on insertion sequence *6110* (IS*6110*). A qPCR assay was designed for detecting a specific region in IS*6110* by BLAST. The IS4 primer/probe concentration, qPCR efficiency and various of PCR additives were evaluated and optimized. Thirty-four species of commonly isolated microorganisms were used for evaluating the analytical specificity. Moreover, 130 clinical specimens were collected for evaluating the performance versus Cobas TaqMan MTB (CTM) assay kit and culture. The amplification efficiencies of IS4 were 99.61% and 102.61% without and with internal control DNA (Bacteriophage Lambda), respectively. Dimethyl sulfoxide outperformed glycerol or BSA for eliciting the most effective amplification and the lowest limit of detection. In evaluating the clinical performance, various specimen types were collected. IS4 demonstrated a high degree of agreement (kappa = 0.71) with CTM. The clinical sensitivity and specificity of IS4 and CTM were 92.11% (35/38), 82.61% (76/92), 84.21% (32/38) and 95.65% (88/92), respectively. The clinical sensitivity and specificity of IS4 were similar for both pulmonary [92.00% (23/25) and 76.92% (30/39), respectively] and extrapulmonary [92.31% (12/13) and 86.79% (46/53), respectively] specimens. Among AFS-negative cases, the clinical sensitivity and specificity remained 90.48% (19/21) and 83.91% (73/87), respectively, with culture as the gold standard. We concluded that IS4, a new primer/probe pair for TaqMan based qPCR assay, was developed, optimized, and validated for the sensitive and specific detection of TB among various specimen types. The performance was not compromised under AFS-negative conditions.

## Introduction

Tuberculosis (TB) infects approximately 10.4 million people and causes 1.4 million deaths annually^1^. TB is one of the top ten causes of death in low-income (6^th^ place) and lower-middle-income (5^th^ place) countries according to an epidemiological report from the World Health Organization (WHO)^1^. It was reported that over 95% of TB-caused deaths occur in low- and middle-income countries. The correct and early diagnosis of TB could elicit the initiation of anti-TB treatments and infection control. It was estimated that 53 million deaths were prevented due to correct anti-TB treatments between 2000 and 2016^1^.

Improvements in TB diagnosis and standardized anti-TB treatments are the keys to the WHO’s End-TB strategy^2,3^. Correct treatments against TB mainly depend on accurate TB diagnosis^4^. Various diagnostic methods, including acid fast stain (AFS), TB culture, nucleic acid hybridization probes, line probe assays, and nucleic acid amplification tests (NAATs), have been developed for TB diagnosis^4–6^. AFS provides the technical advantage of low cost, fast turn-around-time (TAT), and simple implementation. However, this method was reported to have low sensitivity and specificity for TB diagnosis^5^. TB culture is regarded as the gold standard method for TB diagnosis^4,6,7^. In clinical practice, TB culture is normally performed as a back-up test method for AFS^4,5,7^. TB culture is generally believed to be accurate, but the TAT largely depends on the growth rate of TB bacteria, which is regarded as slow-growing mycobacterium. Consequently, the time required for TB diagnosis could be longer than weeks to months, which hinders rapid diagnosis and TB treatment. In contrast, both nucleic acid hybridization probes and line probe assays use oligonucleotide probes to identify differences in target 16S or 23S rDNA for discriminating TB from other non-tuberculosis Mycobacterium (NTM)^5^. These methods have been reported to be accurate and even able to detect rifampicin or isoniazid drug resistance^4^. These methods do not rely on nucleic acid amplification to provide a sufficient number of target molecules for the tests^5^.

To address the above mentioned issues, NAATs were developed for improvement in TAT for TB diagnosis. NAAT methods can achieve superior performance over TB culture-based methods. Several DNA regions, including the intergenic spacer (ITS) from the 16S or 23S rRNA genes, MPB64, insertion sequences *986* or *6110* (IS*986* or IS*6110*), etc., have been selected as NAAT PCR targets^8^. Insertion sequences (ISs) are the smallest and most numerous independently transposable mobile genetic elements widely present in shaping their host genomes, such as eukaryotic and bacterial genomes^9–11^. IS elements carry genes encoding a transposase in their host sequence, which increases their host opportunity to survive in a variety environmental conditions. Genome diversification, deletions, inversions, duplications and other host arrangements have been due to IS mobilization and expansion via transposition^12,13^. *Mycobacterium tuberculosis* contains at least 30 different IS elements in its genome, one of which, IS*6110*, is present in MTB complex (MTBC) members in multiple copies (over 25 copies), except in *Mycobacterium bovis* BCG, in which it appears to be present as a single or fewer copies, and other mycobacteria, where it is absent^14^. Consequently, it has been reported that IS*6110* could be widely used for TB diagnosis in clinical specimens; previous studies have established the validity of the detection rate and sensitivity of MTBC targeting *IS6110*, showing that it was significantly higher than culturing MTB for a long time period^15–18^.

Both commercial and laboratory developed test (LDT) NAATs have been developed based on these target regions. The Roche Cobas TaqMan MTB (CTM) assay which focuses on a segment of the 16S rRNA gene^19^ is one of the most widely used commercial NAATs and it has attained 96% sensitivity and 74% specificity with AFS-positive smears, while the sensitivity and specificity dropped to 64% and 99.3%, respectively, with AFS-negative smears^5,20^. The result indicated CTM assay is good for AFS-positive cases; however, the sensitivity is suboptimal for AFS-negative cases, which is especially difficult for rapid diagnosis and treatment. Besides, despite the fact that manufacturer instruction narrowed CTM application only for the pulmonary specimens, some researchers also evaluated the diagnostic accuracy of CTM for the extrapulmonary specimens. A meta-analysis study analyzing 15 studies indicated that the diagnostic accuracy of CTM for the pulmonary specimens was superior to the extrapulmonary specimens. The summary indicated sensitivity and specificity is 81% and 99% in the pulmonary specimens, while the sensitivity and specificity dropped to 59% and 98%, respectively, for the extrapulmonary specimens^19^. In addition to CTM, the Xpert MTB/RIF assay (Cepheid, Sunnyvale, CA) is another commercialized NAATs, which approved by World Health Organization for rapid TB diagnosis, allows completely automated nuclei acids preparation, amplification, and detection rifampin resistance of *M. tuberculosis* by quantitative PCR with a disposable single test cartridge in the meantime. One study indicated it has attained 100% sensitivity and 67% specificity with AFS-positives cases; whereas those values were 40% and 99%, respectively with AFS-negative cases^21^. Another study also reported that the sensitivity of Xpert MTB/RIF was much higher for AFS-positive disease (99.0%) compared with AFS-negative disease (70.3%)^21^. Moreover, the sensitivity and specificity of Xpert were 79.0% and 97%, respectively in the extrapulmonary specimens when TB culture serves as the reference method^21^. In brief, sensitivity of Xpert MTB/RIF test for extrapulmonary samples was found to be at moderate level; sensitivity of the test was found to be decreased especially in AFS-negative samples. In summary, CTM and Xpert MTB/RIF have consistently shown high sensitivity among AFS- and culture-positive specimens, however their variable sensitivity in AFS-negative or extrapulmonary specimens has raised concern^22,23^. The added benefit of NAATs over AFS could be limited owing to the insufficient sensitivity for AFS-negative cases. Moreover, commercial assays cannot fulfil the needs for testing MTBC in extrapulmonary specimens, which may account for considerable numbers of samples in tertiary teaching hospitals. The cost may be another issue, as the cost would be higher for a commercially available kit than for LDT NAATs, given that most TB-prevalent areas are developing countries^5,6^. Consequently, re-addressing the issue of MTBC NAATs and developing an efficient LDT NAAT is essential.

In this study, we designed a novel quantitative real-time polymerase chain reaction (qPCR)-based NAAT based on IS*6110*. IS*6110* is a MTBC NAAT target region^8^. The discriminating advantage of IS*6110* over other targets is that IS*6110* was almost only identified in MTBC^24,25^. The proposed method was optimized and evaluated with respect to its analytical sensitivity and specificity. Moreover, 130 clinical specimens were collected for method validation. The CTM assay and the proposed method were conducted simultaneously with AFS and culture to evaluate performance.

## Results

### Establishing the primer/probe sets for the multiplex qPCR assay

We developed a new multiplex TaqMan-based qPCR assay to determine MTBC. In addition to designing specific primer/probe sets for MTBC detection, we also designed primer/probe sets for Lambda plasmid DNA, which were used as an internal control in our assay. Three amplicon colours were determined by differences in the peak emission wavelength of the three fluorescent dyes, including 6-FAM-labelled IS4 probe emitting blue, HEX-labelled Lambda DNA probe emitting green, and ROX, a passive reference dye, emitting red. Our main goals for designing primers were a GC content of 50–60%, an optimal melting temperature for the primers ranging from 60–62 °C and a primer length was shorter than 25 bases. The probes are in the middle of the forward and reverse primer binding sites, did not overlap with the primer-binding site on the sense strand and have a Tm 10 °C higher than the primer.

### A highly conserved region inside IS*6110* was chosen for detecting MTBC

**Figure 1:**
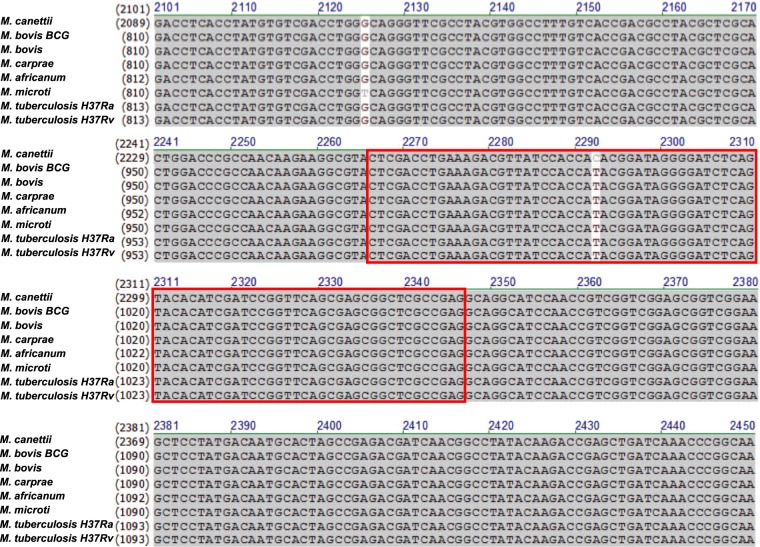
IS*6110* sequence comparison among MTB complex. Identical nucleotides are shown as black letters on a gray background, and similar nucleotides are indicated as black letters on a white background. Red box indicates the region of IS4 qPCR amplicon.

IS*6110* is an insertion sequence that is found exclusively within the MTBC that comprises *M. tuberculosis, M. africanum, M. bovis, M. cannetti, M. caprae, M. microti, and M. pinnipedi*. This element has been recognized as a multi-copy target for the molecular detection of MTBC, which increases the detection sensitivity; this has been mentioned by many reports. In our study, we also chose IS*6110* as our target region for designing the specific primers and probe due to its highly conserved DNA sequence between MTBC. As expected, the IS*6110* DNA sequence shares 99.9% identity with MTBC. The partial IS*6110* nucleotide sequences from MTBC are shown in Fig. 1. Two single nucleotide polymorphisms occur in *M. cannetti* and *M. microti*. The IS4 amplicon and probe binding site are inside the red box (Fig. 1). The results also confirmed that IS*6110* has become an important diagnostic tool in the differentiation of MTBC species from other mycobacteria because it shows a highly conserved sequence. We could not find the *M. pinnipedi* IS*6110* sequence in the NCBI database, so the data is not shown.

### Validation of the qPCR assay with international standards

**Figure 2:**
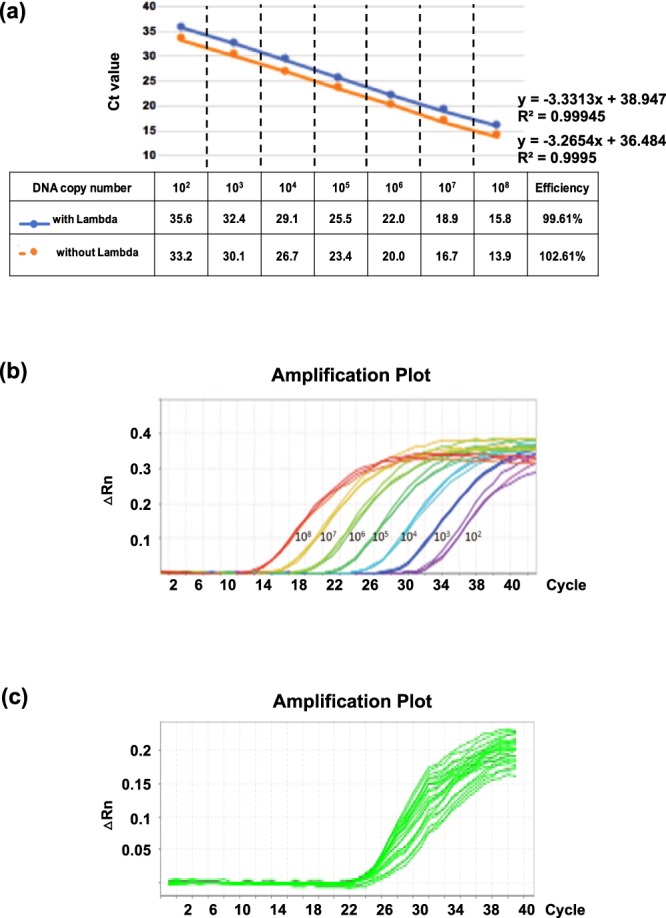
Multiplex quantitative polymerase chain reaction (qPCR) assay using seven samples of which Ld DNA was mixed with 10-fold serial dilutions of IS*6110*-containing synthetic vectors. (**a**) Standard curve. Threshold cycle (y axis) of the reaction is plotted against the Log of *M. tuberculosis* genomic DNA copies (x axis). 10-fold serial dilutions were done from 10^8^ to 10^2^ copies and were tested with (blue) and without (orange) Lambda DNA. The standard curve plot, slope, Ct value, Y-intercept, and R^2^ were shown. (**b**) Detection of IS*6110*-containing synthetic vectors with a FAM-labeled probe (495–520 nm) showing the increasing number of cycles required to detect decreasing amounts of DNA. △Rn (y axis) of the reaction was plotted against the Ct value (x axis). The sample with lowest DNA concentration (10^2^ copies) could be detected with 35 cycles. Each experiment was run in triplicate and the result is reproducible. △Rn means Rn minus the baseline. Rn is the reporter signal normalized to the fluorescence signal of ROX Dye. (**c**) Detection of Lambda DNA synthetic vectors with a HEX-labeled probe (656–662 nm) showed that 30 cycles are required to detect 1,000 copies of Lambda DNA.

To verify the efficiency of real-time PCR amplification, the primer and probe set sensitivity were investigated. The synthetic IS*6110* plasmid DNA, which included the IS4 primer/probe set target region, was obtained at a concentration of 10^8^ genomic DNA copies. The synthetic IS*6110* plasmid DNA was serially diluted 10-fold to 10^2^ genomic DNA copies and were used to construct a standard curve. The assay covered 7 orders of magnitude (i.e., 10^2^, 10^3^, 10^4^, 10^5^, 10^6^, 10^7^ and 10^8^). The results revealed that the IS4 primer efficiency (E) was 99.9%, with a slope of –3.33 and a correlation coefficient (R^2^-value) > 0.99 for the singleplex PCR standard curve (without the Lambda DNA primer/probe sets) (Fig. 2(a)). In contrast, for the multiplex PCR standard curve (with the Lambda DNA primer/probe sets), the IS4 primer efficiency (E) was 102.4%, with a slope of −3.26, and showed a similar correlation coefficient to the IS4 singleplex reaction. The results may indicate that the Lambda DNA primer/probe sets may slightly disturb the IS4 primer/probe. Compared with the IS4 singleplex reaction (Ct values from 13.9 to 33.2), the multiplex results produced amplification curves with relatively higher Ct values (15.8 to 35.6), suggesting a lower sensitivity by the multiplex reaction (Fig. 2(b)). Given that the Lambda DNA primer/probe sets may disturb the IS4 primer efficiency, we spiked a relatively lower Lambda DNA copy (1,000 copies) and decreased the primer/probe concentration. The Ct values under these conditions were approximately 30 (Fig. 2(c)). Briefly, the Lambda DNA primer/probe sets may elicit interference from the IS4 primer/probe sets, but the effect was minor. The efficiency of the IS4 primer/probe sets was high and was also specific for its target region (no other non-specific bands were observed in the agarose gel electrophoresis) (Supplemental Fig. 1).

### Dimethyl sulfoxide (DMSO) improves qPCR sensitivity

**Figure 3:**
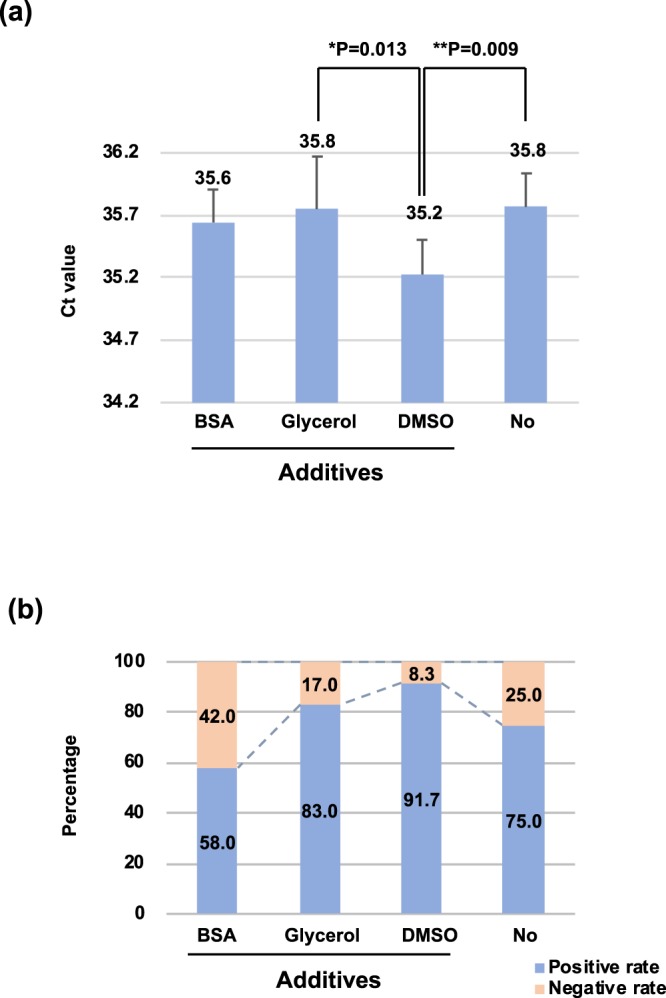
DMSO enhanced qPCR performance. (**a**) The IS4 amplicons were amplified in the presence of BSA (0.8 μg/μl), Glycerol (4%) or DMSO (4%), respectively (initial DNA: 10 copies). The figure listed the average, standard deviation (STD) of Ct value for each treatment. The average Ct values and STD were calculated on the basis of twelve replicates. The average Ct of DMSO treatment was significantly lower than the other treatments. (**b**) IS4 qPCR assay was able to detect 10 copies of IS*6110* synthetic vectors in the presence of different additives. The percentage was shown in the bars. Positive rate means PCR amplification successfully. Negative rate means not detected.

We evaluated various additives (bovine serum albumin (BSA), Glycerol and DMSO) to optimize the analytical sensitivity of the qPCR reaction. Comparison of the glycerol, BSA and no additives, 4% DMSO produced a relatively low Ct value (35.2 ± 0.28) (Fig. 3(a)). A significant difference in the Ct values among the various additives was noted (P = 0.0013). The negative rate was lowest (8.3%) when DMSO served as the additive compared with the other additives (Fig. 3(b)).

### The theoretical limit of detection (LOD) is lower than 10

Ten copies of standard plasmid were used to determine the LOD of the assay. The LOD was confirmed by repeating the qPCR twenty-one times. qPCR with DMSO as additive revealed a positive rate higher than 90%, while the other treatments attained lower positive rates (58% for BSA, 83% for glycerol, and 75% for no additive). The limit of detection (LOD) of the test was estimated to be lower than 10 copies (Fig. 3(b)).

### The best concentration of forward versus reverse primer is two-fold

**Figure 4:**
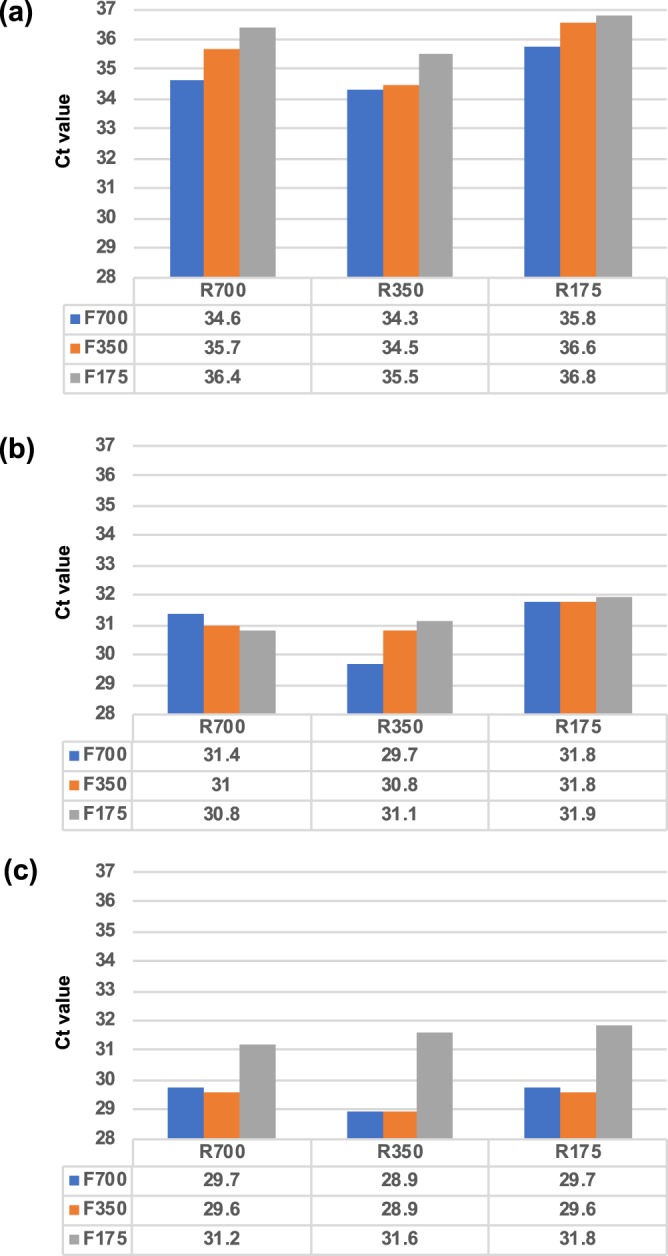
Primer optimization. Various combinations of primers were tested, (**a**) by using 100 copies of DNA amount: combination composed of F700 (Forward primer, 700 nM) & R350 (Reverse primer, 350 nM) attained significantly lowest Ct value. (**b**) by using 1,000 copies of DNA amount: combination composed of F700 (Forward primer, 700 nM) & R350 (Reverse primer, 350 nM) attained significantly lowest Ct value. (**c**) by using 10,000 copies of DNA amount: all the primer combinations generated similar Ct values except F175 combinations, whose forward primer concentration was 175 nM.

We determined the Ct value of different primer concentration combinations to optimize the qPCR conditions. The results indicated that the Ct value was lowest when the forward and reverse primer concentrations were 700 nM and 350 nM, respectively, when the DNA template was 100, 1,000 and 10,000 copies (Fig. 4(a–c), respectively).

### Analytical specificity

Various commonly isolated microorganisms were used for evaluating the analytical specificity. Eleven clinically occurring non-tuberculosis mycobacterium (NTM), seven bacterial species and sixteen fungal species that are commonly reported as causes of respiratory infections clinically were used as quality control samples. All the strains were obtained from the microbiology laboratory at the Chang Gung Memorial Hospital (CGMH) at Linkou (Supplemental Table 1).

### Performance comparison versus CTM and culture

Among the 130 clinical specimens, the number of culture, AFS, CTM assay, and IS4 positive cases were 38, 22, 36, and 51, respectively. We collected clinical specimens to evaluate the performance of the IS4 qPCR assay under three different conditions. Overall, IS4 qPCR showed 16 positive results from 94 samples that were not detected by the CTM assay. The IS4 qPCR showed 16 positive results from the 92 samples regarded as negative MTBC growth by culture. Specifically, IS4 qPCR showed only one positive reaction from the 16 NTM cases, which were proven by culture (see sample No. 90 in Supplemental Table 2), indicating a low rate of cross-reactivity. The degree of agreement (kappa as the index) versus the CTM was 0.71 (95% confidence interval from 0.58–0.83), indicating good agreement. The overall clinical sensitivity and specificity were 92.11% (35/38) and 82.61% (76/92) with respect to culture as the gold standard, respectively (Table 1, panel: overall specimens).

**Table 1:** Performance comparisons between the index test method (IS4), the reference qPCR (CTM assay), and culture under different conditions, including overall specimens, pulmonary specimens (sputum and bronchoalveolar lavage (BAL)), extrapulmonary specimens, AFS-positive specimens and AFS-negative specimens.

	CTM+	CTM−	Culture+	Culture−
**Overall specimens (Sample size: 130)**				
IS4+	35	16	35	16
IS4−	1	78	3	76
CTM+	—	—	32	4
CTM−	—	—	6	88
**On pulmonary specimens (Sample size: 64)**				
IS4+	22	10	23	9
IS4−	1	31	2	30
CTM+	—	—	22	1
CTM−	—	—	3	38
**On extrapulmonary specimens (Sample size: 66)**				
IS4+	13	6	12	7
IS4−	0	47	1	46
CTM+	—	—	10	3
CTM-	—	—	3	50
**On AFS-positive specimens (Sample size: 22)**				
IS4+	17	1	16	2
IS4−	1	3	1	3
CTM+	—	—	16	2
CTM−	—	—	1	3
**On AFS-negative specimens (Sample size: 108)**				
IS4+	18	15	19	14
IS4−	0	75	2	73
CTM+	—	—	16	2
CTM−	—	—	5	85

CTM: Cobas TaqMan MTB assay; AFS: acid fast stain; +: positive; −: negative.

The performance of the IS4 assay versus the CTM qPCR kit and culture was also compared under various specimen conditions. In pulmonary specimens, namely, sputum and bronchoalveolar larvage (BAL) specimens, IS4 showed 10 positive results from 41 cases that were revealed to be negative by the CTM assay. Similarly, the IS4 assay showed 9 positive results from 39 culture negative cases. The clinical sensitivity and specificity were 92.00% (23/25) and 76.92% (30/39) for the pulmonary specimens with respect to culture as the gold standard, respectively (Table 1, panel: on pulmonary specimens). In the extrapulmonary specimens, the IS4 assay showed 6 positive results from 53 cases that were not detected by the CTM assay, while the IS4 assay showed 7 positive results from 53 culture negative cases. The clinical sensitivity and specificity were 92.31% (12/13) and 86.79% (46/53) for the extrapulmonary specimens with respect to culture as the gold standard, respectively (Table 1, panel: on extrapulmonary specimens).

When AFS showed a positive result, the IS4 qPCR assay showed 1 positive among 4 CTM negative cases (Table 1,panel: on AFS-positive specimens, see sample No. 16 in Supplemental Table 2), while the IS4 qPCR assay detected 2 positive results from 5 negative culture cases (Table 1,panel: on AFS-positive specimens, see samples No. 3 and No. 20 in Supplemental Table 2). Among the negative AFS cases, the IS4 qPCR assay showed 15 positive results from 90 cases called negative by the CTM, while the IS4 qPCR assay showed 14 positive results from the 87 negative culture cases. Under the conditions when AFS was negative, the degree of agreement (kappa as the index) versus the CTM was 0.63 (95% confidence interval from 0.46–0.79), which also indicated good agreement. The clinical sensitivity and specificity were 90.48% (19/21) and 83.91% (73/87) with respect to culture as the gold standard, respectively (Table 1, panel: on AFS-negative specimens). Moreover, we summarized the sensitivity and specificity of IS4 and CTM with 95% confidence intervals under various conditions (Table 2).

**Table 2:** Performance summary of the new primer/probe pair (IS4) and the reference qPCR method (CTM assay) under various conditions, including on pulmonary specimens, on extrapulmonary specimens, on AFS-positive specimens, and on AFS-negative specimens.

		Sensitivity (95% CI)	Specificity (95% CI)
Overall specimens	IS4	0.9211 (0.7979–0.9669)	0.8261 (0.7373–0.8887)
	CTM	0.8421 (0.7004–0.9201)	0.9565 (0.8957–0.9807)
On pulmonary specimens	IS4	0.9200 (0.7603–0.9678)	0.7692 (0.6205–0.8697)
	CTM	0.8800 (0.7092–0.9496)	0.9744 (0.8765–0.9872)
On extrapulmonary specimens	IS4	0.9231(0.6882–0.9649)	0.8679 (0.7547–0.9314)
	CTM	0.7692 (0.5132–0.9025)	0.9434 (0.8506–0.9763)
On AFS-positive specimens	IS4	0.9412 (0.7474–0.9724)	0.6000 (0.2684–0.8447)
	CTM	0.9412 (0.7474–0.9724)	0.6000 (0.2684–0.8447)
On AFS0negative specimens	IS4	0.9048 (0.7225–0.9618)	0.8391 (0.7493–0.9002)
	CTM	0.7619 (0.5578–0.8850)	0.9770 (0.9231–0.9906)

CTM: Cobas TaqMan MTB assay; AFS: acid fast stain; 95% CI: 95% confidence interval.

## Discussion

New diagnostic tools are key in the successful fight against TB globally^3^. NAATs are recommended by the WHO as initial tools for cases suspected to have TB. The CDC from the USA also suggested that a NAAT be performed on at least one respiratory specimen with suspected TB^6^. An IS*6110*-based qPCR assay for diagnosing TB was developed in this study. The concentrations of the PCR primers and additives were tested to attain optimal sensitivity. Moreover, clinical specimens were collected for the validation of the qPCR index. The results showed that the index test revealed higher clinical sensitivity than the CTM assay. The index test also showed high performance in both pulmonary and extrapulmonary specimens. Additionally, the index test displayed a high sensitivity when analysing AFS-negative specimens. We developed and validated a high sensitivity qPCR test that may be useful for diagnosing TB in various types of specimens.

IS*6110* is one of the most common regions selected as a PCR target for diagnosing TB^8^. The exclusivity of IS*6110* in MTBC provides a discriminative diagnostic tool for differentiating TB from NTM or other bacteria^26^. However, some NTM strains have been reported to contain specific DNA fragments that are homologous to IS*6110*^27^. Some studies have also shown that IS*6110* primer pairs cross-react with *M. peregrinum* and *M. chelonae*^28^. The cross-reactivity of previously published primers and probes with environmental mycobacteria is common. Consequently, it is necessary to develop a new set of primers and probes for greater detection specificity. To address this issue, we selected the IS*6110* C-terminal region, which has not been chosen by previous reports, as our target. We first blasted the IS4 sequence for different organism categories, including 62 kinds of bacteria, 12 kinds of fungi, and 14 kinds of viruses, according to reference genomic DNA sequence in the NCBI database (data not shown). We also examined the primer specificity by using DNAs from multiple common bacterial, NTM, and fungal species (Supplemental Table 1). Moreover, IS4 showed high specificity in the clinical performance evaluation (Table 1). All of the dry or wet lab results demonstrated that IS4 showed highly specific amplification for detecting MTBC. The frequency of NTM isolation from clinical specimens has significantly increased in several countries^4,29,30^. The symptoms and signs between TB infection and some NTM infection are not always easy to distinguish. Moreover, some important NTMs (e.g., *M. kansasii*) shared similar slow growth rates with TB, which has led to early diagnosis difficulty. AFS can rapidly identify the presence of mycobacteria in clinical specimens. However, AFS displays low sensitivity and cannot distinguish TB from NTM. As a rapid diagnostic tool, IS4 was designed with this issue in mind. The ability of IS4 to distinguish TB from NTM would be an advantageous and essential characteristic in patient management and infection control (Supplemental Tables 1 and 2).

Early diagnosis depends on culture-independent methods, such as AFS and qPCR. The early administration of an anti-TB agent could be initiated once AFS shows a positive result. AFS is a rapid and inexpensive test method; however, it has poor sensitivity. Many true TB infections are not initially detected by AFS. Consequently, the true value of a qPCR assay over other test methods may lie in AFS-negative cases^4,8,31^. qPCR could also provide the added value of detecting paucibacillary disease. Diagnosing paucibacillary disease is essential for clinically diagnosed TB cases that are highly suspected to be infected with TB but all the lab tests fail to provide bacteriological evidence^4^. Equivocal TB cases are considerably frequent in clinical practice because of inappropriate diagnostic methods^6^. Clinically diagnosed TB cases could be troublesome to both physicians and patients owing to the lack of confidence in the administration of anti-TB drugs and isolation policies. The clinical validation study revealed that the IS4 assay was more sensitive than the CTM assay or culture in AFS-negative cases (Table 1, panel: overall specimens). For AFS-negative cases, the IS4 assay detected 14 positives from 87 cases that were not detected by culture. We conducted a medical chart review for these 14 cases (Table 1, panel: on AFS-negative specimens). Among the 14 cases, 2 cases were under treatment with anti-TB drugs (See samples No. 43 and 130 in Supplemental Table 2). Three cases had previously been diagnosed with TB but were currently radiographically stable (See samples No. 68, 117 and 120 in Supplemental Table 2). Three cases displayed an immunocompromised status (See samples No. 43, 64 and 90 in Supplemental Table 2). The IS4 assay may provide valuable hints to both physicians and patients when TB is highly suspected and there is no evidence from traditional test methods. The medical chart review results implied that the IS4 assay could detect minimal bacterial loads in latent TB cases. The high detection sensitivity may provide additional information for physicians in deciding the initiation of anti-TB treatments. In contrast, for AFS-positive specimens, the advantages of the IS4 assay over AFS are its ability to distinguish MTBC from NTMs^4,32,33^.

Several commercial TB diagnostic tools are doing well and widely applied in many countries. However, current commercial TB diagnostic tool, CTM and GeneXpert, are approved for pulmonary specimens and not for extrapulmonary specimens. TB can infect various tissue types beside lung tissues. In this investigation, the IS4 assay showed similar performance in both pulmonary and extrapulmonary specimens (Table 1, panel: on pulmonary and extrapulmonary specimens). In CGMH, TB qPCR requests obtained from extrapulmonary specimens have already accounted for half of all the TB qPCR orders. Additional validation on both pulmonary and extrapulmonary specimens is necessary for both commercial and in-house developed TB qPCR assays. Moreover, GeneXpert TB diagnostic tools can detect TB and drug resistance simultaneously by multiplexing qPCR^34^. This design can enhance the TAT of TB detection and anti-TB resistance detection, which is especially useful in high TB and high drug resistance prevalence settings. In contrast, the positive predictive value of anti-TB drug resistance would be low in a low prevalence setting^4^. The additional cost and time of a test to confirm anti-TB drug resistance adversely suggests the use of a multiplex test in low a TB burden setting. In Taiwan, the resistance rate of new TB cases to rifampicin is 2%^35^.

The IS4 qPCR conditions were optimized by the addition of additives. BSA, glycerol, and DMSO were used and evaluated in this study. All three additives are common materials for enhancing PCR performance. BSA has been reported to enhance the PCR process by stabilizing DNA polymerase^36^. Glycerol is an enzyme-stabilizing solvent and enhances PCR yield through a similar mechanism as BSA^37^. Moreover, DMSO reduces the Tm and the complex secondary structure so that qPCR sensitivity can be enhanced^38–40^. The effects of BSA, glycerol, and DMSO on qPCR efficiency were evaluated and are shown in Fig. 3(a), where PCR with DMSO as an additive showed lower Ct than the other additives. No difference was observed between BSA and glycerol. Moreover, DMSO also outperformed the other additives when studying the LOD (Fig. 3(b)), where the qPCR reaction with DMSO could attain the most stable results (the representative rate is 91.7%) when the initial amount of nucleic acid was very low (e.g., 10 copies in the LOD study). The results implied that the improvement in the qPCR efficacy and the LOD in the IS4 assay may be due to the reduction in the Tm or nucleic acid secondary structures. The possible interactions between primers, probes, and the target template could also be observed in the primer optimization study (Fig. 4). The F700-R700 pair did not attain the lowest Ct value. In contrast, slightly reduced reverse primer (R350) with F700 resulted in the most effective application with lower initial DNA amounts (i.e., 100 copies). The phenomenon could result from the location of the IS4 probe. The IS4 probe is located on the sense strand and close to the forward primer. The location of the IS4 probe may therefore have an effect on the forward primer binding site. The interaction between primers and the target template may also be the reason hindering the optimal yield. DMSO, therefore, is a reasonable additive for reducing these interactions.

There are some limitations in this study that may hinder its wide application in the current setting. First, the IS4 qPCR method was validated at a single tertiary medical centre. The clinical performance of the IS4 qPCR assay should be re-validated in other hospitals, especially those with different epidemiological settings. Second, we only focused on developing new qPCR primers and a probe pair. Specimen pre-processing and DNA purification prior to qPCR analysis should be evaluated on several other instruments that expose a higher risk of cross-contamination^5^. Further work on the development of a closed PCR system (i.e., from-specimen-to-result system) is necessary in the future. Moreover, as a qPCR method, IS4 cannot distinguish live from non-viable MTBC and cannot be used to monitor responses to treatment. AFS, culture, and clinical data are still essential for a more comprehensive diagnosis of TB infection.

## Materials and Methods

### *Mycobacterium tuberculosis* complex strains were used for IS*6110* alignment

The mycobacteria strains were identified by BLAST search in the National Center for Biotechnology Information database (http://www.ncbi.nlm.nih.gov/BLAST/). The strains included *M. canetti* CIPT 140060008, *M. bovis BCG str. Tokyo172*, *M. bovis AF2122/97*, *M. carprae* strain Allgaeu, *M. africanum* GM041182, *M. microti* str. 12, *M. tuberculosis* H37Ra and *M. tuberculosis* H37Rv.

### Sequence alignment among *Mycobacterium tuberculosis* complexes (MTBCs)

The deduced IS*6110* nucleotide sequences were aligned by Vector NTI 9.0 software (Invitrogen, Carlsbad, CA)

### qPCR additive evaluation

PCR additives have been used to enhance the signal and increase the yield, specificity and consistency of PCR reactions. These additives may have different effects on PCR amplification under particular conditions. We chose three different functional additives, including DMSO, BSA and glycerol, in our study. Given that GC content of IS*6110* is 62%, DMSO was chosen for this GC-rich template, which may promote DNA double strand dissociation, reduce the secondary structures of the IS4 primer/probe sets, and prevent primer/target mis-priming.

### Real-Time PCR Analysis

Each multiplex PCR assay was performed in a 30 µl final reaction volume containing 15 µl of 2X QuantiNova Probe PCR mastermix (Qiagen, Hilden, Germany), 1.5 µl ROX passive reference dye (an inert additive that provides a constant fluorescent signal for sample normalization, which was provided by Applied Biosystems), 0.2 μl of 100 pmol/μl IS4F (forward primer for the target region), 0.2 μl of 50 pmol/μl IS4R (reverse primer for the target region), 0.8 μl of 1pmol/μl IS4P (probe for the target region), 9 µl of DNA (10^8^-10^2^ genome copies), 1.2 µl of 100% DMSO (Alfa Aesar 36480AP), 0.4 µl of 10 pmol/μl LdF (forward primer for Lambda) and LdR primers (reverse primer for Lambda), 0.2 μl of 10 pmol/μl LdP (probe for Lambda), 1 μl of Lambda DNA (as spiked DNA, 1,000 genome copies) and 0.1 μl of nuclease-free water.

The following thermal profile was used with the Applied Biosystems StepOne real-time PCR system (Applied Biosystems, Carlsbad, CA): (1) an initial step of 2 min at 95 °C and (2) 40 cycles of 5 s at 95 °C and 10 s at 60 °C. A negative control with double-distilled water was performed for each real-time PCR experiment.

### Primer and probe design

All of the probes and primers were synthesized by Integrated DNA Technologies, Inc. (http://www.idtdna.com). The primer and probe design was based on the homology region of the partial insertion sequence *6110* (IS*6110*) in the *Mycobacterium tuberculosis* complex (MTBc) for all species. A 141 base pair region, which represents one of the most highly conserved regions, was chosen as the target region for primer amplification. The sense and antisense primers and double quenched probe were designated IS4F, IS4R, and IS4P, respectively. The fluorogenic IS4P probe labelled with a unique fluorescent reporter dye [6-carboxyfluorescein (FAM)] at the 5′-end, an internal ZEN Quencher and a 3′-Iowa Black Fluorescent Quencher (IBFQ), was designed to anneal to an internal sequence in the amplified region. A 48 kb-Lambda DNA plasmid (Genbank) isolated from bacteriophage lambda (cI857*ind* 1 *Sam* 7) was chosen as an internal control to check the qPCR process. The sense and antisense primers used to amplify the Lambda DNA were manually designed to amplify a 111 bp amplicon inside the 34,000-35,000 region, which has no conserved sequence in the BL21(DE3) *E. coli* strain and were designated LdF and LdR, respectively. The probe was covalently labelled with 4,4,7,2′,4′,5′,7′-hexachloro-6-carboxyfluorescein (HEX) at the 5′-end, an internal ZEN Quencher and a 3′-Iowa Black Fluorescent Quencher (IBFQ) at the 3′-end and was designated LdP. The primer was designed using the online PrimerQuest Tool on IDT website (https://sg.idtdna.com/PrimerQuest/Home/ Index). All the primers, probes used for real-time PCR have listed in Table 3.

**Table 3:** Primers and TaqMan probes sequences of IS4 qPCR assay.

Target	Forward primer (5′-3′)	Reverse primer (5′-3′)	Probe (5′-3′)	Amplicon (bps)
IS4	CTCGACCTGAAAG ACGTTATCC	CTCGGCTAGTGCA TTGTCATA	AGTACACAT/ZEN/ CGATCCGGTTCAAGCG	141
Lambda	AGCACTGTAAGGT CTATCG	CCTGTTGGTTGGG GTAAG	ACCGCCCTA/ZEN/ TTCTCTCGCTGA	111

### Clinical specimens

The clinical specimens requested for the TB PCR test were obtained from individuals suspected of having TB (judging by clinical physicians, according to their clinical symptoms/signs^6^). The types of pulmonary specimens included expectorated sputum, bronchoalveolar lavage. The types of extrapulmonary specimens consisted of pus, tissue, and pleural effusion. The specimens were enrolled consecutively between Oct 2017 and Jan 2018 at the Department of Laboratory Medicine, Chang Gung Memorial Hospital at Linkou. For the enrolled cases, AFS and TB cultures were also ordered by the caring clinical physicians. All the clinical specimens were collected according to the diagnosis needs of the daily practice and not specifically for the study. The authors declare this study was approved by the Chang Gung Medical Foundation Institutional Review Board (IRB no. 201701364B0) and granted a waiver of patient consent because no patient at risk.

### Specimen processing

AFS and culture for the detection of mycobacteria were ordered together for each specimen. For sputum and pus specimens, N-acetyl-L-cysteine and NaOH were mixed with specimens for mucolysis and decontamination, respectively. The mixture was incubated for 15 minutes after adequate mixing. After incubation, the mixture was centrifuged with 3420 g at 20 °C for 15 minutes. The supernatant was discarded and the sediment was collected for the smear (1 cm × 2 cm). Phosphate buffer was added to neutralize the remaining sediment for culture. In contrast, for fluid specimens (i.e., BAL, CSF, pleural effusion, and ascites), the specimen was centrifuged under 3420 g at 20 °C for 15 minutes. The supernatant was discarded and phosphate buffer was added to neutralize the remaining sediment. For tissue specimens, the specimen was cut into small pieces, followed by the addition of 1 ml 0.9% saline. The supernatant was taken for the smear and culture. The clinical specimens in suspicious of TB were processed in biosafety level (BSL)-3 area.

### Acid Fast Stain

The processed specimens were spread on a slide for the smear (1 cm × 2 cm). A smear was completely covered by 0.3% Ziehl-Neelsen carbofuchsin (Wako Pure Chemical Industries Ltd., Osaka, Japan), followed by adequate heating for 5 seconds. The smear was incubated at room temperature for 5 minutes. The smear was then rinsed with sterile water (ddH_2_O) followed by 3% acid alcohol. Subsequently, the smear was rinsed again with sterile water (ddH_2_O), followed by the addition of 0.3% methylene blue onto the smear for 25 seconds. The AFS test was performed in BSL-3 area.

### Mycobacteria culture

We performed cultures in both solid and liquid media. The processed specimen was dropped onto Lowenstein Jensen (LJ) Media (Creative Life Sciences Inc., New Taipei City, Taiwan) and the LJ media was incubated at 36 °C and 5% CO_2_. A small volume (0.5 ml) of the processed specimen was added into Mycobacteria Growth Indicator Tubes (MGIT) and incubated with the BD BACTEC™ MGIT™ system (Becton Dickinson, MD, USA). A subculture was created in 7H11 media (Creative Life Sciences, New Taipei City, Taiwan), followed by positive growth in MGIT. A single colony obtained from either LJ media or 7H11 media was collected for species identification. We performed preliminary species identification of mycobacteria based on the growth rate and colony texture. An immunochromatographic test (TAUNS Laboratories Inc., Shizuoka, Japan) was used for confirming the MTBC. For the isolates whose growth rates and colony textures did not suggest MTBC, a MicroFlex LT mass spectrometer (Bruker Daltonik GmbH, Leipzig, Germany) was used to identify the NTM species. We observed the growth status of both LJ media and MGIT for 8 weeks. Negative mycobacterium growth was reported when no growth was noted within 8 weeks. The TB culture was performed in BSL-3 area.

### Nucleic acid extraction

DNA was extracted from the specimens before nucleic acid amplification for either the proposed qPCR method (i.e., IS4) or the reference qPCR. For sputum and pus specimens, 300 μl of the specimen were mixed with 330 μl lysis buffer and 30 proteinase K, followed by incubation at 56 °C for 10 minutes for inactivation. For tissue specimens, the specimen was cut into small pieces and then 1 ml 0.9% saline was added, followed by incubation at 56 °C for 10 minutes. All the specimens were processed in BSL-3 area until inactivation. The nucleic acid extraction and PCR assay were performed in BSL-2 condition. The nucleic acids were isolated from 300 μl of the inactivated mixture and the DNA was extracted into 60 μl elution buffer using a LabTurbo nucleic acids Extraction Kit (TaiGen Biotechnology Inc., Taiwan) according to the manufacturer’s specifications.

### Reference qPCR (CTM assay)

The extracted DNA was analysed by the CTM assay to serve as the reference qPCR. The amplifier mix was prepared by adding 200 μl of magnesium reagent and 50 μl of an internal control to 460 μl of master mix. Fifty μl of this amplifier mix was added to 50 μl of the DNA extraction solution. This mix was used to perform amplification and detection with an MTB TaqMan analyser. The MTB-positive sample and positive control Ct values were recorded. Twenty copies of the MTB gene were contained in the TaqMan MTB positive control (as determined by personal communication with the manufacturer). The quantity of MTB can therefore be determined by calculating the difference between the positive control Ct value (Ct/p) and that of the sample (Ct/s) using the following formula: Number of MTB bacilli ¼ [2^(Ct/p − Ct/s)] × 20. All the reagents were provided by the manufacturer.

### Statistical analysis

Analysis of variance (ANOVA) was used to determine the Ct values between the treatments with the various PCR additives. We used Tukey’s honest significant difference post hoc test for further comparison when the null hypothesis of ANOVA was rejected. Graph Pad Prism 5.0. was used for the statistical analysis and a p value < 0.05 was considered statistically significant. The 95% confidence intervals of sensitivity and specificity of IS4 and CTM were estimated by the method proposed by Agresti and Coull^41^.

## Electronic supplementary material

Supplementary Information
