# Supplementary material for: Development of a high sensitivity TaqMan-based PCR assay for the specific detection of Mycobacterium tuberculosis complex in both pulmonary and extrapulmonary specimens
Source: Sci Rep. 2019 Jan 14;9:113. doi: 10.1038/s41598-018-33804-1 (PMC6331544; doi:10.1038/s41598-018-33804-1)
Supplement: Supplementary file 1 — Supplementary Information [file 41598_2018_33804_MOESM1_ESM.pdf]

A proposed paper submitted to
Scientific Reports
Development of a high sensitivity TaqMan-based PCR assay for the specific detection of Mycobacterium tuberculosis complex in both pulmonary and extrapulmonary specimens

Hsin-Yao Wang[1,2]†, Jang-Jih Lu[1,3,4]†, Ching-Yu Chang[3], Wen-Pin Chou[5], Jason Chia-Hsun Hsieh[6], Chien-Ru Lin[5]*, Min-Hsien Wu[5,6]**

[1]Department of Physical Medicine & Rehabilitation, Chang Gung Memorial Hospital at Linkou, Taoyuan City, Taiwan

[2]Ph.D. Program in Biomedical Engineering, Chang Gung University, Taoyuan City, Taiwan

[3]Department of Medical Biotechnology and Laboratory Science, Chang Gung University, Taoyuan City, Taiwan

[4]School of Medicine, Chang Gung University, Taoyuan, Taiwan

[5]Graduate Institute of Biomedical Engineering, Chang Gung University, Taoyuan, Taiwan

[6]Taiwan / Division of Haematology/Oncology, Department of Internal Medicine, Chang Gung Memorial Hospital at Linkou, Taoyuan City, Taiwan

† Hsin-Yao Wang and Jang-Jih Lu contributed equally to this manuscript

****Corresponding author**: Min-Hsien Wu, Ph.D.

Graduate Institute of Biochemical and Biomedical Engineering, Chang Gung University, Taoyuan, Taiwan / Division of Haematology/Oncology, Department of Internal Medicine, Chang Gung Memorial Hospital at Linkou, Taoyuan City, Taiwan

Tel.: +886-3-2118800 ext 3599

Fax: +886-3-2118668

E-mail: mhwu@mail.cgu.edu.tw

***Corresponding author**: Chien-Ru Lin, Ph.D.

Graduate Institute of Biochemical and Biomedical Engineering, Chang Gung University, Taoyuan, Taiwan

Tel.: +886-3-2118800 ext 3213

Fax: +886-3-2118668

E-mail: crl0608@mail.cgu.edu.tw

Keywords: TaqMan real-time PCR; Mycobacterium tuberculosis

Supplemental Figure 1. qPCR products specificity was checked using 2% agarose gel electrophoresis.

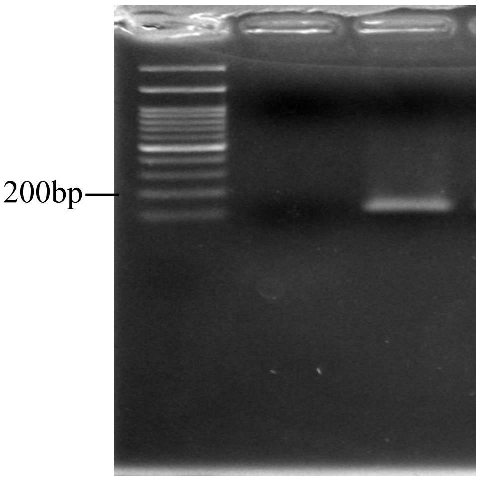

The Lane 1,2 and 3 are cropped from the same gel.

Lane 1: DNA marker, NEB 100bp; Lane 2: NTC; Lane 3: the qPCR product.

Supplemental Table 1. Species distribution of the samples used for IS4 specificity test, comprised of three categories: Non-tuberculosis Mycobacteria (NTM), bacteria and Fungus. n means different batch of sample preparation and test.

Species		n
NTM	*M. abscessus*	7
	M. avium	2
	M. chimaera-intracellulare group	4
	M. cosmeticum	1
	M. farcinogene senegalense group	1
	M. fortuitum	1
	M. gordonae	1
	M. mageriitum	1
	M. peregrinum	1
	M. kansasii	4
	M. szulgai	1
Bacteria	*A.baumannii*	1
	B-Strepto.Gr.B	1
	E.coli	1
	E. faecium	1
	K. pneumoniae	1
	P. aeruginosa	1
	S. epidermidis	1
Fungus	*A. flavus*	1
	A. fumigatus	1
	A. niger	1
	A. terreus	1
	C. albicans	1
	C. glabrata	1
	C. guilliermondii	1
	C. krusei	1
	C. lusitaniae	1
	C. neoformans	1
	C. parapsilosis	1

Supplemental Table 1. (Continue)

Fungus	*C. tropicalis*	1
	F. solani	1
	S. apiospermum	1
	S. prolificans	1
	T. asahii	1

Supplemental Table 2. Comparative analysis of clinical diagnosis and isolation of mycobacteria from different samples derived from patients with AFB Smear, Culture, CTM and IS4 Assay. a: 1~2 acid fast bacilli/300field, b: acid fast bacilli 1+, c: acid fast bacilli 2+, d: acid fast bacilli 3+, e: acid fast bacilli 4+, P: positive, N: negative, SP: sputum, BAL: broncho-alveolar lavage, CSF: Cerebrospinal fluid, AS: Ascites, PL: Pleural effusion, TS: Tissue, PC: Pericardial fluid, OTH: Other, MTBC: mycobacteria tuberculosis complex, CT: ct values by qPCR, CTM: Cobas TaqMan MTB kit.

No.	Specimen	CTM Ct	CTM result	IS4 Ct	IS4 result	Culture result	AFS result
1	SP	N	N	N	N	*M.avium*	N
2	SP	47.6	P	N	N	MTBC	a
3	PUS	45.3	P	34.7	P	N	a
4	SP	N	N	N	N	MTBC	N
5	TS	N	N	N	N	MTBC	N
6	SP	N	N	N	N	*M.peregrinum*	N
7	SP	44.8	P	34.9	P	MTBC	d
8	PL	N	N	41.3	P	MTBC	N
9	SP	N	N	N	N	*M.abscessus*	N
10	SP	N	N	N	N	*M. chimaera* intracellulare group	N
11	PL	N	N	N	N	*M.kansasii*	N
12	SP	N	N	N	N	*M.kansasii*	d
13	BAL	N	N	N	N	*M.kansasii*	N
14	BAL	N	N	N	N	*M.abscessus*	N
15	SP	N	N	N	N	*M.abscessus*	a
16	SP	N	N	41	P	MTBC	a
17	SP	N	N	N	N	*M.abscessus*	a
18	PUS	42.7	P	33.2	P	MTBC	b
19	PL	N	N	36.4	P	MTBC	N
20	PUS	39.5	P	36.3	P	N	b
21	TS	N	N	N	N	*M.cosmeticum*	N
22	BAL	N	N	N	N	*M.abscessus*	N

Supplemental Table 2. (Continue)

23	SP	N	N	N	N	*M. chimaera* intracellulare group	N
24	PL	45.1	P	38.3	P	MTBC	N
25	TS	35.8	P	31	P	MTBC	N
26	BAL	39.5	P	34	P	MTBC	N
27	PL	49.3	P	39.4	P	MTBC	N
28	TS	40.4	P	34.4	P	MTBC	b
29	SP	31.1	P	31	P	MTBC	e
30	PUS	35.5	P	31.6	P	MTBC	e
31	BAL	38.7	P	34.4	P	MTBC	c
32	BAL	36.6	P	32.8	P	MTBC	b
33	BAL	44.9	P	37.4	P	MTBC	N
34	PUS	38.6	P	34.7	P	MTBC	b
35	SP	33.7	P	28.5	P	MTBC	c
36	SP	43.6	P	36.5	P	MTBC	c
37	BAL	38	P	32.4	P	MTBC	b
38	SP	40	P	27.8	P	MTBC	N
39	SP	43	P	36.4	P	MTBC	N
40	SP	36.2	P	30.6	P	MTBC	a
41	SP	45.5	P	35.5	P	MTBC	a
42	TS	41.8	P	36.7	P	MTBC	b
43	PUS	33	P	28.4	P	N	N
44	SP	41.2	P	35.4	P	MTBC	N
45	SP	40	P	30.6	P	MTBC	N
46	SP	33.6	P	28.6	P	MTBC	N
47	BAL	37	P	30.1	P	MTBC	N
48	BAL	40.8	P	33.3	P	MTBC	N
49	SP	39.9	P	32.8	P	MTBC	e
50	SP	35.9	P	30.8	P	MTBC	N
51	SP	43.6	P	32	P	MTBC	N
52	CSF	43.1	P	35.6	P	MTBC	N
53	BAL	N	N	N	N	N	N

Supplemental Table 2. (Continue)

54	SP	N	N	N	N	N	N
55	SP	N	N	29.4	P	MTBC	N
56	TS	N	N	N	N	N	N
57	SP	N	N	N	N	*M.szulgai*	N
58	TS	N	N	N	N	N	N
59	PL	N	N	N	N	N	N
60	BAL	N	N	N	N	N	N
61	BAL	N	N	N	N	N	N
62	CSF	N	N	N	N	N	N
63	CSF	N	N	N	N	N	N
64	BAL	N	N	37.2	P	N	N
65	TS	N	N	37.2	P	N	N
66	BAL	N	N	N	N	N	N
67	BAL	N	N	N	N	N	N
68	BAL	N	N	36.8	P	N	N
69	TS	N	N	N	N	N	N
70	PL	N	N	N	N	N	N
71	BAL	N	N	N	N	N	N
72	CSF	N	N	N	N	N	N
73	AS	N	N	N	N	N	N
74	SP	N	N	36	P	N	N
75	BAL	N	N	N	N	N	N
76	FTS	N	N	N	N	N	N
77	BAL	N	N	37	P	N	N
78	TS	N	N	N	N	N	N
79	TS	N	N	N	N	N	N
80	TS	N	N	N	N	N	N
81	TS	N	N	N	N	N	N
82	SP	N	N	N	N	N	N
83	BAL	N	N	N	N	N	N
84	BAL	N	N	37	P	N	N
85	TS	N	N	N	N	N	N

Supplemental Table 2. (Continue)

86	PL	N	N	N	N	N	N
87	TS	N	N	N	N	N	N
88	BAL	N	N	36.9	P	N	N
89	PL	N	N	N	N	N	N
90	TS	N	N	36.9	P	*M. abscessus*	N
91	OTH	N	N	N	N	N	N
92	PL	N	N	N	N	N	N
93	TS	N	N	N	N	N	N
94	CSF	N	N	N	N	N	N
95	BAL	N	N	N	N	N	N
96	CSF	N	N	N	N	N	N
97	CSF	N	N	N	N	N	N
98	TS	N	N	N	N	N	N
99	SP	N	N	N	N	N	N
100	TS	N	N	N	N	N	N
101	OTH	N	N	N	N	N	N
102	AS	N	N	N	N	N	N
103	BAL	N	N	N	N	N	N
104	AS	N	N	36.4	P	N	N
105	PL	N	N	N	N	N	N
106	CSF	N	N	N	N	N	N
107	TS	N	N	N	N	N	N
108	TS	N	N	N	N	N	N
109	BAL	N	N	N	N	N	N
110	CSF	N	N	N	N	N	N
111	SP	N	N	N	N	N	N
112	TS	N	N	N	N	N	N
113	BAL	N	N	N	N	N	N
114	CSF	N	N	N	N	N	N
115	CSF	N	N	N	N	N	N
116	TS	N	N	36.7	P	N	N
117	BAL	N	N	36.1	P	N	N

Supplemental Table 2. (Continue)

118	PL	N	N	N	N	N	N
119	BAL	N	N	N	N	*M. chimaera* intracellulare group	N
120	BAL	N	N	36.1	P	N	N
121	PC	N	N	N	N	N	N
122	AS	N	N	N	N	N	N
123	AS	N	N	N	N	N	N
124	PL	N	N	N	N	N	N
125	BAL	N	N	N	N	N	N
126	PUS	N	N	N	N	N	N
127	PL	N	N	N	N	N	N
128	CSF	N	N	N	N	N	N
129	TS	39.3	P	34.2	P	MTBC	N
130	BAL	41.2	P	35.3	P	N	N
